# RELATIONSHIP BETWEEN WORKING ENVIRONMENT AND CARE AIDES’ COMPASSION FATIGUE, BURNOUT AND COMPASSION SATISFACTION

**DOI:** 10.1093/geroni/igae098.1814

**Published:** 2024-12-31

**Authors:** Ashikur Rahman, Yinfei Duan, Carole Estabrooks

**Affiliations:** University of Alberta, Edmonton, Alberta, Canada; University of Alberta, Edmonton, Alberta, Canada; University of Alberta, Edmonton, Alberta, Canada

## Abstract

Severe staff shortages, sustained stress, low compassion satisfaction, high compassion fatigue, and serious levels of burnout among health care workers are frequently reported in the literatures. Limited research has examined compassion-related work life among long-term care (LTC) aides facing high-stress situations due to the deaths of residents and colleagues during COVID-19. In a cross-sectional study with 760 care aides working in 28 LTC homes in Alberta, Canada, we used a two-level multilevel regression analysis to examine how working environments were related to compassion fatigue, burnout, and compassion satisfaction using the Professional Quality of Life (ProQOL-9) scale. Care aides in smaller facilities reported higher levels of compassion fatigue (B = 0.764, p = 0.008) compared to those in larger facilities. Higher compassion satisfaction (B = 1.009, p = < 0.001) and lower burnout (B = -0.909, p = < 0.001) were observed when care aides perceived more supportive working cultures. Care aides reported higher compassion fatigue when there was a lack of structural (B= -0.149, p =0.019) or staffing resources (B= -0.253, p = 0.007). We also found that not having enough staff (B = -0.469, p = < 0.001) or enough time (B = -0.337, p= 0.019) to complete tasks was significantly associated with higher levels of burnout. In conclusion, compassion fatigue and burnout among care aides were associated with some modifiable factors in the working environment. These findings offer direction on which elements of the working environment may be promising for improvement efforts.

